# Data on characteristics of reported cases of unilateral posterior reversible encephalopathy syndrome (PRES)

**DOI:** 10.1016/j.dib.2019.104648

**Published:** 2019-10-11

**Authors:** Tadashi Ozawa, Ryota Tanaka, Risa Nagaoka, Yuhei Anan, Younhee Kim, Kosuke Matsuzono, Takafumi Mashiko, Reiji Koide, Haruo Shimazaki, Keisuke Ohtani, Yusuke Amano, Kensuke Kawai, Shigeru Fujimoto

**Affiliations:** a Division of Neurology, Department of Internal Medicine, Jichi Medical University, Japan; b Department of Neurosurgery, Jichi Medical University, Japan; c Department of Diagnostic Pathology, Jichi Medical University, Japan

**Keywords:** Posterior reversible encephalopathy syndrome, Unilateral distribution, Brain, Vasogenic edema

## Abstract

Data presented in this article are related to our article entitled “Unilateral posterior reversible encephalopathy syndrome: A case report” [1]. Cases of Posterior Reversible Encephalopathy Syndrome (PRES) involving unilateral lesions are very rare. We searched the PubMed database using keywords such as PRES, unilateral, and asymmetric and found a small number of cases to include in our review. We summarized the characteristics of these reported cases of unilateral PRES, including our case.

**Table:** Specifications Table

Subject	Clinical Neurology
Specific subject area	Posterior reversible encephalopathy syndrome
Type of data	Table
How data were acquired	Cases were collected from Pubmed database and summarized directly.
Data format	Raw, analyzed.
Parameters for data collection	The cases of unilateral PRES were collected with PubMed database.
Description of data collection	We searched the PubMed database using keywords PRES, unilateral, and asymmetric.
Data source location	Division of Neurology, Department of Internal Medicine, Jichi Medical University School of Medicine, Tochigi, Japan
Data accessibility	Data are within this article.
Related research article	T Ozawa, R Tanaka, R Nagaoka, Y Anan, Y Kim, K Matsuzono, T Mashiko, R Koide, H Shimazaki, K Ohtani, Y Amano, K Kawai, S Fujimoto, Unilateral posterior reversible encephalopathy syndrome: A case report., Clin. Neurol. Neurosurg. 185, 2019, 105493 [1].

**Table:** 

**Value of the Data** • These data contribute to further knowledge of Neurology by reporting the rare cases of unilateral posterior reversible encephalopathy syndrome. • The data can provide the courses and symptoms of few reported cases with unilateral posterior reversible encephalopathy syndrome. • The data show the diversity of posterior reversible encephalopathy syndrome, which supports to consider the pathogenesis.

## 1 Data

The data presented in this report were analyzed based on our case and the eight cases of unilateral PRES we confirmed via our PubMed search [[1], [2], [3], [4], [5], [6], [7], [8]]. Our search yielded two other cases, but those contained no description of the patients' condition, and consequently were excluded from our analyses [9]. We described hypertension that was thought to be related to the development of PRES and the lesions observed in these cases. In addition, the possible causes of PRES confirmed from the reports that we reviewed were described (see Table 1).

**Table 1:** Patient and characteristics of reported cases of unilateral Posterior Reversible Encephalopathy Syndrome (PRES).

Author/Year	Age/Sex	History of Hypertension	Blood Pressure at onset	Location of edema	Related factors of unilateral PRES
Huijgen et al., 2014 [2]	36 Female	None	mean arterial pressure 108	entire left hemisphere	subarachnoid hemorrhage, coiling of the left anterior communicating artery aneurysm
Nishijima et al., 2015 [3]	41 Male	Yes	210/125	right supratentorial white matter, brainstem and bilaterally cerebellum	stenosis of the left internal carotid artery, chronic renal failure
Dhar et al., 2011 [4]	47 Female	None	mean arterial pressure 120	left posterior temporal plus parietal	subarachnoid hemorrhage, contralateral vasospasm
Romano et al., 2011 [5]	58 Male	Yes	220/NA	left parietal-occipital lobes	left hyperplastic AChA, mild anemia, hypertension
Çamlıdağ et al., 2015 [6]	49 Female	None	140/90	left front-temporal lobes	left MCA occlusion, renal failure, tacrolimus lung transplantation, mild anemia chronic renal failure, hypertension, tacrolimus, epilepsy
Lee et al., 2008 [7]	18 Female	Yes	>200/NA	right occipital lobe	
Lee et al., 2008 [7]	42 Male	None	198/124	left parietal-occipital lobes	metastatic sarcoma, chemotherapy, alcoholism
Sato et al., 2016 [8]	79 Male	None	123/74	entire right hemisphere	ventriculo-peritoneal shunt for normal pressure hydrocephalus, subarachnoid hemorrhage chronic renal failure, hypertension
Ozawa et al., 2019 [1]	73 Male	Yes	147/83	left front-parietal lobes	

AChA, anterior choroidal artery; MCA, middle cerebral artery; NA, data not available.

## 2 Experimental design, materials, and methods

We searched PubMed for case reports of unilateral PRES in English or Japanese. We then collected data from cases of unilateral PRES reports that we were able to confirm a clear description and imaging. First, we selected the keywords PRES and unilateral and found twenty-four references. After reviewing the content of each document, we confirmed nine cases of unilateral PRES [2,[4], [5], [6], [7], [8], [9]]. We then performed a search using the keywords PRES and asymmetric and found eleven references. We also reviewed these and confirmed three cases of Unilateral PRES [[3], [4], [5]]. Two cases were duplicates of our first search [4,5], which left us with ten cases of confirmed PRES. However, two cases lacked descriptions of the patients' conditions and were consequently excluded from our analyses. Thus, we summarized the features of the remaining eight cases in addition to our case.
